# Tai Chi exercise and neuroplasticity: a narrative review according to neural mechanisms and clinical utilizations in brain health

**DOI:** 10.3389/fnins.2026.1769779

**Published:** 2026-02-19

**Authors:** Xiaoqiang Jin, Juanjuan Chen, Xiaoqi Zhang

**Affiliations:** 1 College of Physical Education, Hebei Normal University, Shijiazhuang, China; 2 College of Physical Education, Kaili University, Kaili, China; 3 College of Physical Education and Health Sciences, Guangxi Science and Technology Normal University, Liuzhou, China

**Keywords:** cognitive function, neuroplasticity, neurorehabilitation, physical and mental intervention, TC

## Abstract

**Background:**

Neuroplasticity is the core process by which the brain responds to aging, learning, and injury. Reporting positive non-pharmacological intervention approaches to promote neural plasticity is a core focus of contemporary neuroscience and rehabilitation medicine. Tai Chi (TC), as a traditional Chinese physical and mental practice that deeply combines soothing body movements, breathing regulation, and spiritual focus, is increasingly attracting attention from the scientific community for its role in facilitating brain health.

**Objective:**

Our review seeks to combine recent evidence, elucidate how TC promotes neural plasticity via multi-level mechanisms, discuss its advantages in promoting cognitive, motor, and emotional functions, and investigate its clinical utilization prospects and future research challenges in neurorehabilitation.

**Methods:**

According to reviewing recent literature, we combined evidence from cross-sectional studies, randomized controlled trials, systematic reviews, and meta-analyses, with a center on citing research findings utilizing multimodal neuroimaging techniques (such as fMRI, fNIRS, EEG) and molecular biology techniques to construct a complete chain of evidence from molecules to systems.

**Findings:**

TC drives multi-level neural plasticity modifications via its unique physical and mental combination properties. At the macro level, it can enhance the gray matter volume of the hippocampus and prefrontal cortex, and promote the organizational effectiveness of large-scale functional networks in the brain. At the micro molecular level, TC establishes a favorable microenvironment for neuronal survival, synaptic plasticity, and neural repair by upregulating BDNF, increasing endogenous antioxidant defense, modulating inflammatory balance, and improving mitochondrial energy metabolism. These structural, functional, and molecular level changes collectively form the neurobiological basis for TC to promote memory and executive function, increase balance and motor management, and promote emotional regulation ability. Our review further assesses the clinical effectiveness of TC in the rehabilitation of neurological diseases, such as Parkinson’s disease (PD), stroke, and mild cognitive impairment, determining that it not only decreases symptoms, but may also have the possible role to decrease disease development. Ultimately, our review delve into the challenges and future perspectives experienced by this range in the context of standardization of research paradigms, causal reasoning of mechanisms, and individualized interventions.

## 1 Introduction

The human brain exhibits remarkable plasticity via the lifespan—an inherent capacity to continuously reshape its structure and function in response to experience, learning, and environmental interactions (Marzola et al., 2023; Statsenko et al., 2025). This dynamic adaptive mechanism is a core tenet of modern neuroscience, acting as the foundation not only for skill acquisition and memory formation, but also as a vital process for coping with aging and neurological disorders. Nevertheless, in contemporary society, the prevalence of age-associated cognitive decline, neurodegenerative diseases, and neurological deficits following stroke constitutes a significant public health challenge. Hence, the search for non-pharmacological intervention approaches that can positively, safely, and widely improve neuroplasticity to sustain brain health and accelerate neural repair has turned into an urgent research focus.

Among different interventions, physical exercise is broadly determined as a potent means of driving neuroplasticity (Erickson et al., 2011). Emerging evidence, nevertheless, reveals that the type of exercise is critical; “mind–body exercises,” which deeply combine physical activity with mental regulation, may confer unique advantages for the brain beyond those of conventional exercise via synergistic effects (Rosso et al., 2025; Babiy et al., 2025). In this context, Tai Chi (TC) — a traditional Chinese mind–body practice that, along with other modalities such as yoga and mindfulness meditation, emphasizes the integration of physical activity with regulated breathing and mental focus. Unlike the predominantly static postures of yoga, TC is characterized by continuous, slow, and flowing movement sequences, presenting a unique challenge for dynamic balance and motor-cognitive coordination (Yeung et al., 2018). This unique multimodal combination elevates it beyond mere physical activity, rendering it a continuous challenge for sensorimotor combination, cognitive control, and emotional regulation.

Although evidence is accumulating to encourage the advantages of TC for promoting balance, muscle strength, cognitive function, and emotional wellbeing (Solloway et al., 2016; Wehner et al., 2021; Yue et al., 2020), the scientific understanding of its potential neurobiological processes – notably how it regulates neuroplasticity – remains in its nascent stages. Recent research is commonly fragmented across behavioral, imaging, or molecular levels, lacking a unified framework that combines these multi-level findings. While previous reviews have effectively mapped the health outcomes of TC (Solloway et al., 2016) or summarized its benefits in specific populations, a comprehensive, multi-level synthesis linking its behavioral effects to underlying neuroplasticity mechanisms is lacking.

Therefore, Our review seeks to elucidate how TC, as a complex mind–body intervention approach, promotes neuroplasticity across numerous dimensions, from the molecular and systems levels to the behavioral level. We will first analyze how the combined mind–body characteristics of TC constitute a unique stimulus for neuroplasticity. We combine recent evidence to suggest the remodeling of brain structure and functional networks driven by TC and detail its manifestations in motor, cognitive, and emotional functions. Moreover, we investigate the potential molecular processes potential the neuroprotective impacts of TC, assess its practical effectiveness in the rehabilitation of particular neurological diseases, and explore future research perspectives and challenges in the field. By constructing this integrative evidence chain, spanning from microscopic processes to macroscopic utilizations, we seek to offer an authoritative interpretation of the neuroscience principles of TC and promote its scientific utilization in clinical and public health settings.

## 2 Methods

### 2.1 Literature search strategy

This narrative review employed a comprehensive and systematic search strategy designed to capture the broad spectrum of evidence linking Tai Chi (TC) to neuroplasticity and multidimensional health outcomes. The goal was to maximize sensitivity, ensuring the inclusion of studies utilizing diverse methodological approaches, from molecular assays and multimodal neuroimaging to behavioral and clinical assessments. We systematically searched four major electronic databases: PubMed, Web of Science Core Collection, Scopus, and PsycINFO in December 2024. The search timeframe spanned from the inception of each database to December 2024, encompassing both foundational and recent research. The search strategy was constructed using a multi-concept, Boolean logic framework, combining controlled vocabulary (e.g., MeSH in PubMed, Emtree in Embase) with free-text terms. The strategy was developed iteratively, informed by existing high-quality systematic reviews in related fields and reviewed by a collaborator with expertise in molecular physiology to ensure comprehensiveness of the molecular/physiological terms. The core concepts and their representative search terms (illustrated using PubMed syntax) are summarized in Table 1.

**Table 1:** The core concepts and their representative search terms.

Concept group	Core objective	Example search terms (PubMed)
1. Intervention	Identify all studies on Tai Chi/Qigong.	“Tai Ji”[MeSH] OR “Tai Chi”[TW] OR Taiji[TW] OR “Tai Chi Chuan”[TW] OR Qigong[MeSH/TW]
2. Mechanism/measurement	Capture literature on neuroplasticity, all relevant neuroscientific measures, and key molecular pathways.	Neuroplasticity: “Neuroplasticity”[MeSH] OR “Neuronal Plasticity”[TW] OR “Brain Plasticity”[TW]
		Neuroimaging (Structural & Functional): “Neuroimaging”[MeSH] OR “Magnetic Resonance Imaging”[MeSH] OR “Diffusion Tensor Imaging”[MeSH] OR “DTI”[TW] OR “voxel-based morphometry”[TW] OR “cortical thickness”[TW] OR “Electroencephalography”[MeSH] OR “EEG”[TW] OR “Magnetoencephalography”[TW] OR “MEG”[TW] OR “Near-Infrared Spectroscopy”[MeSH] OR “fNIRS”[TW] OR “Positron-Emission Tomography”[MeSH] OR “PET”[TW]
		Molecular & Physiological: “Brain-Derived Neurotrophic Factor”[MeSH] OR “BDNF”[TW] OR “Oxidative Stress”[MeSH] OR “Antioxidants”[MeSH] OR “Inflammation”[MeSH] OR “Cytokines”[MeSH] OR “Interleukin-6”[TW] OR “Tumor Necrosis Factor-alpha”[TW] OR “Heart Rate”[MeSH] OR “Heart Rate Variability”[TW]
3. Outcome	Include studies measuring cognitive, motor, emotional, and psychosocial effects.	Cognitive: “Cognition”[MeSH] OR “Memory”[MeSH] OR “Executive Function”[TW] OR “Attention”[MeSH]
		Motor & Balance: “Postural Balance”[MeSH] OR “Gait”[MeSH] OR “Motor Skills”[MeSH] OR “Accidental Falls”[MeSH] OR “balance”[TW]
		Emotional & Psychosocial: “Emotional Regulation”[TW] OR “Affect”[MeSH] OR “Depression”[MeSH] OR “Anxiety”[MeSH] OR “Stress, Psychological”[MeSH] OR “Quality of Life”[MeSH]

### 2.2 Literature screening and selection process

The screening process was conducted independently by two reviewers (X. J. and J. C.) using Covidence systematic review software. Any discrepancies were resolved through consensus or, when necessary, adjudication by a third senior reviewer (X. Z.). The selection process followed the PRISMA (Preferred Reporting Items for Systematic Reviews and Meta-Analyses) guidelines, as detailed in the flow diagram. De-duplication: Automated and manual removal of duplicate records. Title/Abstract Screening: Records were screened against pre-defined, broad eligibility criteria: (a) involving human subjects of any age/health status; (b) investigating Tai Chi or Qigong as a primary intervention; (c) reporting on outcomes related to brain structure/function, neuroplasticity mechanisms, or relevant cognitive/motor/emotional/clinical outcomes. Full-Text Review: Potentially eligible articles were retrieved and assessed in full.

Final Inclusion Criteria: We prioritized randomized controlled trials (RCTs) and longitudinal intervention studies. To construct a rich, contextual narrative, we also included: High-quality cross-sectional studies (e.g., comparing long-term practitioners with naive controls). Relevant systematic reviews and meta-analyses to inform background and discussion. Exclusion Criteria: Non-English literature (due to resource constraints), conference abstracts without full data, unpublished theses, and studies where Tai Chi was part of an inseparable multi-component intervention.

### 2.3 Data synthesis and analytical framework

Given the narrative and integrative aim of this review, a formal quantitative meta-analysis was not performed. We adopted a thematic synthesis approach. Extracted data (study design, population, intervention details, key findings across biological, imaging, and behavioral levels) were organized into a coherent matrix. Our analytical framework was designed to trace evidence across levels of analysis: Molecular/Cellular Level (e.g., BDNF, oxidative stress, inflammation). Systems/Network Level (e.g., brain structure, functional connectivity, electrophysiology). Behavioral/Clinical Level (e.g., cognitive test scores, balance metrics, psychological scales). We explicitly evaluated the level of evidence (e.g., RCT vs. cross-sectional) and methodological strengths/limitations of included studies (e.g., sample size, control condition, blinding). These considerations are woven into the narrative to provide a critical appraisal of the evidence base and to clarify the distinction between correlation and causation where appropriate.

## 3 Findings

### 3.1 TC and neuroplasticity

#### 3.1.1 Characteristics of TC: combination of physical movement and psychological regulation

TC, as a traditional Chinese mind–body exercise with a long history, is fundamentally defined by the organic combination of slow, fluid physical movements with deep breathing regulation and mental focus (Yeung et al., 2018). It is not only a physical activity, but a integrative practice that stresses “the unity of form and spirit” and “the combination of body and mind” (Yeung et al., 2018). The movements highlight continuity, circularity, and lightness, consistently needing practitioners to sustain correct body posture, high levels of mental concentration, and internal qi balance throughout the dynamic process (Qu et al., 2024). This unique mind–body combination model elevates TC beyond conventional exercise, rendering it a practice that significantly promotes emotional stability and mental tranquility, enabling practitioners to face a significant state of mind–body combination during movement.

Long-term TC practice confers multifaceted advantages for physical health. A systematic review and meta-analysis determined that TC training effectively increases muscle strength (e.g., grip strength), physical endurance (e.g., six-minute walk distance), static balance ability (e.g., eyes-open single-leg standing time), and trunk flexibility (Wehner et al., 2021). Concurrently, the positive impacts of TC on mental health are substantiated by wide evidence. An evidence shaping study determining that, across many clinical and research domains, TC mechanisms substantial randomized controlled trial (RCT) evidence encouraging its improvement of psychological wellbeing, health in older adults, balance, and cognitive function (Solloway et al., 2016).

The potential process involves TC’s capacity to effectively regulate the autonomic nervous system via the combination of physical movement, deep and slow breathing, and mental meditation (Lu and Kuo, 2014). During practice, the decrease in respiratory rate and the state of concentration promote the restoration of homeostasis, decrease stress responses linked to the hypothalamic–pituitary–adrenal (HPA) axis, and alter the balance of the autonomic nervous system toward parasympathetic dominance, hence decreasing mental stress and anxiety (Yeung et al., 2018). The impact of TC on emotion regulation is hypothesized to involve networks similar to those engaged by other mind–body practices. Mechanistic insights from related fields, such as mindfulness meditation, suggest a role for the prefrontal cortex (e.g., in top-down regulation), limbic system (e.g., amygdala reactivity), and striatum in emotion regulation, as well as modulation of inflammatory and stress-related gene expression (Tang et al., 2015; Creswell et al., 2019). While direct neuroimaging and molecular evidence specific to TC for all these pathways is still emerging, the shared emphasis on attentional control and present-moment awareness between TC and mindfulness practices provides a plausible basis for this hypothesis (Chiesa and Serretti, 2010). Direct TC studies have shown alterations in prefrontal activity (e.g., increased oxygenation and functional connectivity) (Qi et al., 2023; Chen et al., 2020) and prefrontal-limbic connectivity (Liu et al., 2018), supporting this view. Furthermore, preliminary TC studies indicate it can reduce systemic inflammation (Liu et al., 2022) and upregulate neurotrophic factors like BDNF (Sungkarat et al., 2018), offering molecular parallels to mechanisms observed in contemplative neuroscience. While physical activity alone promotes the brain, its tight combination with psychological activities, such as mindfulness and breathing regulation drives more significant neural effects, synergistically facilitating structural and functional remodeling of brain networks, together with adaptive improvement, ultimately obtaining the goal of increasing overall brain function and health (Rosso et al., 2025; Babiy et al., 2025).

#### 3.1.2 Fundamental concepts of neuroplasticity: structural and functional changes

Neuroplasticity is the fundamental capacity of the brain to dynamically alter and adapt its neural structure and function in response to experience, learning, or injury (Marzola et al., 2023; Statsenko et al., 2025). This concept has revolutionized the traditional view of the adult brain as a static organ, implying its possible role for developing improvement and remodeling throughout the lifespan (Marzola et al., 2023; Statsenko et al., 2025). According to its manifestations, neuroplasticity is mainly categorized into two distinct types: structural plasticity and functional plasticity.

##### 3.1.2.1 Structural plasticity

Structural plasticity denotes physical modifications in the nervous system at the microscopic and even macroscopic levels. This includes modifications in the strength and number of connections between neurons (synapses), and the generation of new neurons (neurogenesis) within particular brain regions (La Rosa et al., 2020). A core process potential this mechanism is activity-dependent synaptic pruning: during progression or learning, the nervous system selectively strengthens frequently utilized neural connections while eliminating seldom-utilized or inefficient synapses according to patterns of neural activity, hence refining and increasing the effectiveness of neural circuits (Faust et al., 2021). Such structural improvement constitutes the physical basis for encoding new memories and forming complex skills. For example, a seminal study has reported that aerobic exercise training can directly increase hippocampal volume and promote spatial memory in older adults, exemplifying the capacity of experience to drive positive structural modifications in the brain (Erickson et al., 2011).

##### 3.1.2.2 Functional plasticity

Functional plasticity displays the brain’s ability to adapt and recover following injury (e.g., stroke, traumatic brain injury) via functional reorganization (Nakamura et al., 2009). When a particular brain region is damaged, its functions are not necessarily permanently lost (Nudo, 2013). Instead, the brain can compensate through two main approaches: (1) redistributing functions from the damaged area to undamaged regions within the ipsilateral or contralateral hemisphere, and (2) increasing the functional effectiveness of remaining healthy neural circuits (Chen et al., 2010). Modern neuroimaging techniques, such as functional magnetic resonance imaging (fMRI), can visually capture this mechanism of post-injury functional reorganization, offering vital evidence for rehabilitation diagnosis and prognostic evaluation (Chen et al., 2010).

Via its unique mind–body combined practice, TC concurrently engages and promotes both structural and functional plasticity in the brain (Cui et al., 2019). In healthy individuals, TC functions as a complex sensorimotor and cognitive challenge that continuously induces the improvement of neural circuits. For instance, it can improve the functional specialization of brain networks; increased brain functional specialization driven by TC practice is a significant predictor of greater cognitive flexibility (Cui et al., 2021). Under the backdrop of neurological rehabilitation, TC can cause the brain toward advantageous functional reorganization. For example, in patients with Parkinson’s disease, TC training has been displayed to significantly promote postural stability, walking capacity, and overall motor function, with impacts even surpassing those of conventional resistance or stretching exercises (Li et al., 2012). This reveals that TC, as a behavioral intervention, can effectively cause adaptive modifications in the brain, hence encouraging the nervous system in obtaining maintained enhancements in both function and structure over the long term.

### 3.2 Impact of TC on neural structure

#### 3.2.1 The impact of TC on brain structure: modifications in the hippocampus and prefrontal cortex

**Figure 1:**
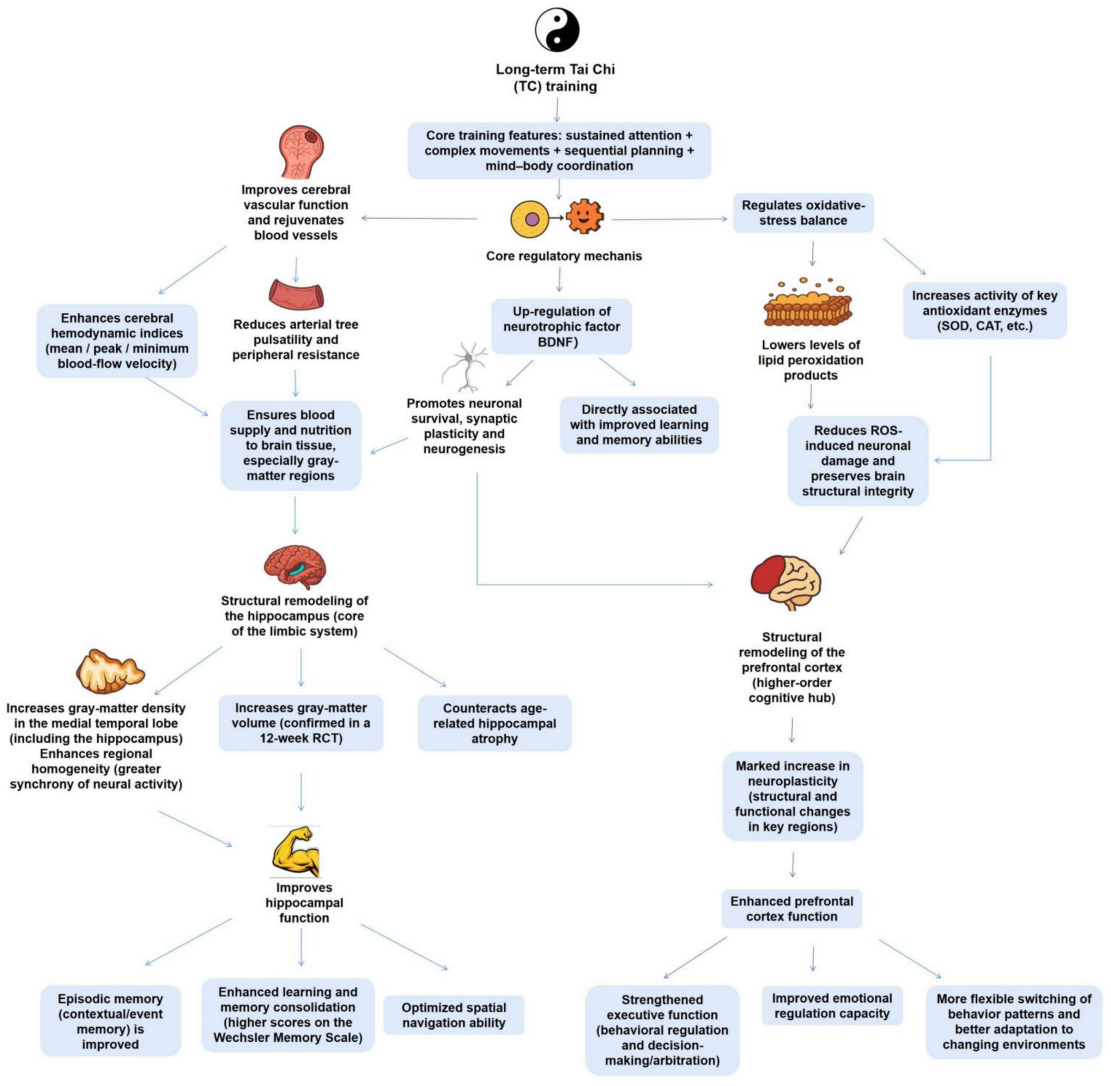
The regulatory mechanism of TC on the structural modification and cognitive function of hippocampus and prefrontal cortex. This figure illustrates the core logic of how long-term TC training affects brain structure and cognitive function: TC training with the core features of “sustained attention+complex motor sequence planning+mind body coordination” provides a structural plasticity basis for the hippocampus and prefrontal cortex through three synergistic mechanisms (improving cerebrovascular function to achieve “vascular rejuvenation,” upregulating BDNF, and regulating oxidative stress balance). In the hippocampus (the core of the limbic system), TC training can increase gray matter density and volume, enhance homogeneity of regional neural activity, combat age-related atrophy, and improve episodic memory, learning memory consolidation, and spatial navigation abilities; In the prefrontal cortex (higher-order cognitive center), TC training enhances neural plasticity, strengthens executive function, emotional regulation, and environmental adaptability.

Long-term TC training can drive significant positive impacts on brain structure, notably in regions critically involved in higher-order cognitive functions, such as memory, emotion regulation, and executive control. Among these, the hippocampus and prefrontal cortex represent some of the most prominent sites for observable plastic modifications (Figure 1).

##### 3.2.1.1 Impact of TC on the hippocampus

The hippocampus, a core component of the limbic system, is vital in learning, memory consolidation, and spatial navigation (Voss et al., 2011). Cross-sectional studies suggest that older women with long-term TC practice exhibit significantly better episodic memory performance in comparison to their peers took part in brisk walking. It is worth noting that magnetic resonance imaging (MRI) data determine that the TC group displays higher gray matter density in the medial temporal lobe (including the hippocampus) and significantly enhanced regional homogeneity in the hippocampus, implying greater synchronization of neural activity within this structure (Yue et al., 2020). These structural and functional modifications are effectively linked to superior memory performance, strongly implying that TC may promote memory by remodeling the structure and function of the hippocampus (Yue et al., 2020). This finding is further encouraged by interventional research: a 12-week RCT displayed that both TC and Baduanjin practice significantly enhanced gray matter volume in memory-associated brain regions (such as the medial temporal lobe and hippocampus) in older adults, together with enhancements in Wechsler Memory Scale scores (Tao et al., 2017). Collectively, these findings determine that TC, as a mind–body exercise, can effectively counteract age-associated hippocampal atrophy, hence decreasing memory decline.

##### 3.2.1.2 Impact of TC on the prefrontal cortex

Beyond the hippocampus, the prefrontal cortex—a higher-order center for executive function, decision-making, and emotion regulation—also displays important structural and functional adaptive modifications in response to TC practice (Yao et al., 2021). Review literature has reported that anatomical and functional brain modifications in TC practitioners are mainly centered in the prefrontal cortex (Yao et al., 2021). The prefrontal cortex is responsible for the executive control of behavior and decision arbitration, enabling flexible switching between exploiting recent behavioral patterns and investigating new approaches in altering environments (Domenech and Koechlin, 2015). The maintained attention, complex motor sequence planning, and mind–body coordination needed during TC practice constitute a continuous challenge and training for the prefrontal executive control functions. This similarly drives increased neuroplasticity in this region, offering a neural structural basis for the enhancements in cognitive control and emotional control linked to TC.

##### 3.2.1.3 Synergistic actions of numerous processes

The potential processes potential TC-driven structural modifications in these brain regions are multifaceted. TC can significantly ameliorate age-associated decline in cerebrovascular function (Li et al., 2021; Zheng et al., 2019). Research displays that long-term TC practitioners exhibit significant enhancements in cerebral hemodynamic indicators, comprising mean blood flow velocity, maximum and minimum blood flow velocities, alongside positive decreases in vascular pulse wave velocity and peripheral resistance. This is equivalent to a “vascular rejuvenation” for the brain, determining adequate blood supply and nutrition to brain tissue, notably to metabolically demanding gray matter regions (Li et al., 2021). Moreover, molecular-level studies implicate core neurotrophic factors. A RCT involving older adults with mild cognitive impairment uncovered that a 6-month TC training regimen not only significantly promoted their memory and executive function, but also concurrently upregulated plasma levels of brain-derived neurotrophic factor (BDNF) (Sungkarat et al., 2018). BDNF is a critical molecule encouraging neuronal survival, synaptic plasticity, and neurogenesis, and its enhanced levels are directly related to enhancements in learning and memory (Azman and Zakaria, 2022). Furthermore, TC can cause a neuroprotective environment by regulating oxidative stress balance. A meta-analysis determined that regular TC practice significantly increases the activity of core antioxidant enzymes, such as superoxide dismutase and catalase, while effectively decreasing levels of lipid peroxidation products (Rosado-Pérez et al., 2021). This helps decrease damage to neurons from reactive oxygen species, hence protecting the structural integrity of the hippocampus and prefrontal cortex via another critical pathway.

#### 3.2.2 Brain network remodeling via the Lens of neuroimaging: functional connectivity and electrophysiological evidence

Developed neuroimaging techniques offer a non-invasive and intuitive window for implying TC-driven neuroplasticity. Moving beyond mere structural analysis, technologies, such as functional magnetic resonance imaging (fMRI) and electroencephalography (EEG) uncover distinctive brain network activity patterns and characteristics of efficient information processing in TC practitioners at the level of dynamic functional organization and neural electrical activity.

Resting-state fMRI evidence has uncovered that long-term TC training can remodel the organization of large-scale functional brain networks. A cross-sectional study comparing the lateralization of brain function between experienced TC practitioners and novices uncovered that the TC group exhibited decreased homotopic functional connectivity in the middle frontal gyrus (Chen et al., 2020). This decrease in connectivity is not indicative of functional decline, but is instead interpreted as a marker of increased neural effectiveness—reflecting increased functional specialization of relevant brain regions and decreased costs for unnecessary interhemispheric information transfer, which along with the superior cognitive performance uncovered in this group (Wang and Lu, 2022). It is worth noting that the duration of practice was negatively linked to homotopic connectivity in the precentral gyrus and precuneus, implying that the neural remodeling impacts of TC follow a dose-dependent relationship, whereby long-term practice continuously improves the functional specialization of brain areas involved in motor planning and self-referential processing (Chen et al., 2020).

This improvement of functional networks is also evident in higher-order cognitive domains. A study by Liu et al. (2018), centering on the neural mechanisms of emotion regulation, uncovered that older adults with long-term TC practice exhibited significantly weaker resting-state functional connectivity between the dorsolateral prefrontal cortex and the middle frontal gyrus in comparison to a control group (Liu et al., 2018). Mediation analysis further suggested that this weakened functional connection served as a complete mediator for the effect of the mindfulness trait “non-judging of inner experience” on emotion regulation ability. This determining that TC may refine emotional balance by streamlining connectivity within the executive control network, enabling more precise and efficient processing of emotional information and avoiding over-regulation, hence obtaining superior emotional equilibrium (Liu et al., 2018).

At the level of neural electrophysiology, EEG studies offer direct evidence for the improved brain functional state driven by TC. A study involving university students found that 15 weeks of TC training significantly enhanced sensorimotor rhythm power (Cha et al., 2024). It should be noted that this pilot study had a relatively small sample size, a common limitation in early-stage mechanistic investigations, which necessitates cautious interpretation and calls for replication in larger cohorts (Cha et al., 2024). Electrophysiological studies suggest that TC practice modulates oscillatory dynamics in a manner indicative of enhanced neural efficiency and adaptive cognitive-emotional states. The observed increase in frontal-midline theta (Fmθ) power is consistently associated with sustained attention, working memory engagement, and cognitive control across various tasks (Cavanagh and Frank, 2014). This aligns with TC’s demand for continuous movement sequencing and mental focus. The enhancement of alpha power (particularly in the sensorimotor and occipital cortices) is not merely a marker of relaxation but is thought to reflect active inhibitory processing, facilitating sensory gating, resource allocation, and internal attention (Jensen and Mazaheri, 2010; Klimesch, 2012). This may underpin TC’s role in reducing distractibility and promoting a state of calm alertness. Concurrently, the reduction in high-frequency beta power may signal a decrease in excessive cortical excitability or “cortical idling” linked to rumination and anxious arousal (Engel and Fries, 2010; Kropotov, 2016). Thus, TC-driven EEG changes likely represent a shift toward a brain state optimized for effortful yet focused task engagement, characterized by efficient top-down control (theta), strategic inhibition of irrelevant input (alpha), and dampened maladaptive high-frequency activity (beta).

This shifts the brain toward a more stable state and significantly increases attentional capacity (Cha et al., 2024). This finding was further corroborated in the particular stressful context of the COVID-19 pandemic. A RCT by Wang and Lyu (2024) displayed that a 12-week simplified TC program not only significantly promoted executive inhibitory function in university students, but also driven favorable modifications in prefrontal theta and alpha wave power (Wang and Lyu, 2024). The TC group performed better on the Stroop incongruent task and exhibited significantly enhanced theta wave activity in both the left and right prefrontal cortices. Frontal theta activity is typically linked to maintained cognitive effort and working memory updating, and its enhancement reveals promoted neural processing effectiveness. In contrast, the control group displayed a significant decrease in prefrontal alpha power, potentially implying the negative impact of pandemic-associated stress on the brain’s baseline state of relaxation. TC training effectively counteracted this adverse effect, sustaining or even increasing the brain’s foundational state for relaxation and concentration (Wang and Lyu, 2024).

Beyond describing regional activation and connectivity changes, a critical question is how the brain computationally instantiates the remodeling observed with TC. The convergence of evidence points toward mechanisms aligned with principles of neural efficiency and network optimization (Lindenberger and Lövdén, 2019). The observed “streamlining” of functional connectivity can be interpreted through the lens of Hebbian plasticity—“neurons that fire together, wire together” (Feldman, 2012). TC’s repetitive, coordinated demands may selectively strengthen task-relevant pathways while pruning less efficient connections. This is reflected in the increased “small-worldness” of functional networks, which balances specialized processing with efficient global communication (Bassett and Bullmore, 2006; Bullmore and Sporns, 2012). At the oscillatory level, enhanced theta-alpha synchronization may facilitate long-range communication between distant brain regions (Fries, 2015; Buzsáki and Draguhn, 2004), while the modulation of oscillatory power could reflect a shift in the excitation-inhibition (E-I) balance within cortical microcircuits, promoting more stable network dynamics (Sohal and Rubenstein, 2019; Buzsáki and Wang, 2012). In essence, TC may act as a repeated challenge that drives the brain toward a more cost-effective and adaptive configuration (Lindenberger and Lövdén, 2019).

Overall, multimodal neuroimaging evidence collectively paints a coherent picture: TC training does not simply increase or diminish brain activity. Instead, it drives significant remodeling of the brain’s functional organization by facilitating functional specialization, improving network connection effectiveness, and driving electrophysiological rhythms that are conducive to cognition and emotion regulation. This synergistic improvement, spanning from macroscopic networks to microscopic electrical activity, constitutes direct evidence for TC’s role in encouraging neuroplasticity and increasing brain function.

### 3.3 Impact of TC on neural function

#### 3.3.1 Multidimensional improvement of motor control, balance, cognition, and emotion by TC

As a mind–body practice combining physical movement, breath regulation, and mental focus, TC confers health benefits spanning numerous levels, from physiological function to higher-order psychological processes. Systematic research displays that long-term TC training significantly increases motor control, balance, cognitive performance, and emotional stability.

##### 3.3.1.1 Increased muscle strength and promoted balance, effectively decreasing fall risk

The enhancement in motor function via TC is first manifested in the maintenance and improvement of muscle strength. A cross-sectional study comparing lower limb muscle strength between 205 long-term TC practitioners and their non-practicing peers uncovered that the TC group exhibited significantly superior isometric strength in the iliopsoas, quadriceps, tibialis anterior, and hamstring muscles in comparison to the control group (Zhou et al., 2016). Notably, no significant differences in muscle strength were uncovered across various age cohorts (60–69, 70–79, 80–89 years) among the practitioners, and muscle strength effectively linked to practice duration, strongly implying that TC effectively mitigates age-associated decline in muscular strength (Zhou et al., 2016). This improvement in strength directly translates to promoted balance and decreased fall risk. A meta-analysis incorporating 24 RCTs offers robust evidence for this effect. The findings determined that TC significantly decreases the risk of falls (Relative Risk: 0.76) and the number of falls in older adults, while also effectively promoting different balance metrics, comprising Timed Up and Go test performance, Functional Reach Test results, and single-leg stance duration (Chen et al., 2023). Subgroup analysis further suggested that TC is positive for both healthy older adults and those at high risk of falls, with its advantages enhancing with longer session duration and higher frequency of practice. Moreover, Yang-style TC displayed superior impacts in comparison to Sun-style (Chen et al., 2023).

##### 3.3.1.2 Remodeling brain networks and increasing cognitive function

Beyond its physical benefits, TC significantly promotes cognitive function, notably in domains linked to the frontal and temporal lobes. An activation likelihood estimation meta-analysis, combining 18 functional neuroimaging studies, uncovered that TC practitioners exhibited consistent functional activity modifications in the superior frontal gyrus in comparison to other forms of exercise or intervention. This suggests that long-term neuroadaptive modifications driven by TC form the neural basis for its cognitive benefits (Wang et al., 2025). This cognitive improvement is tightly related to the remodeling of particular neural circuits. Research uncovered that after a 12-week intervention of either TC or Baduanjin practice, older participants displayed significant enhancement in memory quotient alongside significantly enhanced resting-state functional connectivity between the bilateral hippocampus and the medial prefrontal cortex (Tao et al., 2016). It is worth noting that the degree of improvement in hippocampo-prefrontal functional connectivity was effectively linked to the extent of memory improvement across all participants, offering direct evidence that TC exerts its cognitive protective impacts by improving this vital memory circuit (Tao et al., 2016). Furthermore, functional near-infrared spectroscopy studies centering on TC Zhan Zhuang—a core meditative component of TC—displayed a significant enhancement in oxyhemoglobin concentration within the bilateral prefrontal cortex (including the dorsolateral and ventrolateral regions) during practice, alongside increased functional connectivity between the left and right prefrontal cortices. The brain’s functional network also exhibited more efficient small-world properties (Qi et al., 2023). This determining that TC increases higher cognitive abilities, such as executive control, by augmenting neural activity within and improving the network organization of the prefrontal cortex.

##### 3.3.1.3 Modulating emotion and the autonomic nervous system to promote mental health

The beneficial effects of TC on autonomic nervous system regulation (e.g., increased heart rate variability) and inflammatory profiles are shared with other mind–body practices like yoga (Zou et al., 2018). This suggests common pathways via stress reduction and neuro-immune modulation. However, the specific sensorimotor and attentional demands of TC’s continuous movement may engage brain networks involved in motor planning and executive control more intensely, potentially leading to distinct patterns of neural plasticity. In the realm of emotion regulation, TC has been built as an positive psychological intervention. Review studies determine that TC and Qigong can effectively modulate mood and decrease symptoms of anxiety and depression (Yeung et al., 2018). The potential processes operate at numerous levels: psychologically, the emphasis on “mind–body combination” and mindfulness practice in TC fosters promoted self-awareness and non-judgmental acceptance, hence increasing emotional control capacity; physiologically, its slow, deep breathing and relaxed movement patterns regulate the autonomic nervous system, increasing parasympathetic nervous activity and counteracting stress responses triggered by daily pressures, hence restoring a state of psychosomatic harmony (Yeung et al., 2018).

#### 3.3.2 Relevant evidence: validation of the therapeutic effect of TC in the elderly population and PD rehabilitation

The increasing clinical evidence has built the clear value of TC as an positive non pharmacological intervention in promoting the overall health of the elderly population and decreasing the progression of particular neurological diseases. Its effectiveness has been repeatedly validated in RCTs and long-term follow-up studies.

##### 3.3.2.1 Integrative advantages for the elderly population

For the vast elderly population, TC displays a multidimensional facilitating impact on physical and mental health. On a psychological level, its advantages are notably significant. A systematic review and meta-analysis targeting middle-aged and elderly individuals displayed that TC can significantly promote depressive symptoms, and the most ideal effect was obtained when the intervention period exceeded 24 weeks, the total exercise duration exceeded 2,400 min, and the utilization of 24 simplified TC exercises was utilized (Zeng et al., 2023). Another network meta-analysis compared the therapeutic impacts of different types of TC on anxiety and depression symptoms in elderly people, and uncovered that Yang TC had the best effect in relieving anxiety, while integrative TC exercises performed the best in promoting depression symptoms (Kuang et al., 2024). The possible physiological mechanism of its emotional regulation effect is tightly linked to the improvement of autonomic nervous system function. A meta-analysis on mind body exercises (TC/Yoga) uncovered that these exercises significantly improved heart rate variability parameters, manifested as a decrease in normalized low-frequency power and an enhancement in normalized high-frequency power, while effectively decreasing perceived stress levels (Zou et al., 2018). This reveals that TC may increase the body’s ability to cope with stress by regulating the balance between the sympathetic and vagus nerves, hence exerting an emotional enhancement effect (Zou et al., 2018). Overall, TC promotes emotional health from both psychological and physiological perspectives by combining attention anchoring to bodily sensations, deep and slow breathing, and soothing exercises.

##### 3.3.2.2 Symptom improvement and neuroprotective possible role for Parkinson’s disease

TC has been reported to effectively decrease core motor symptoms in PD patients. An early systematic review and meta-analysis displayed that TC significantly promoted motor function, balance, and functional mobility in PD patients in comparison to conventional care or no exercise (Yang et al., 2014). It is worth noting that current studies have suggested the long-term advantages of TC for PD and its potential biological processes. A one-year RCT uncovered that long-term TC training was even better than brisk walking in promoting step width and Berg Balance Scale scores (Li et al., 2022). The mechanism of promoting balance is linked to increasing the visual network function of the brain and downregulating the inflammatory cytokine interleukin-1 *β*; The overall enhancement in motor function is linked to the improvement of default mode network function, improvement of amino acid and energy metabolism, and the enhancement in mRNA levels of Huntington protein interacting protein 2 in the blood, which may decrease the vulnerability of dopaminergic neurons (Li et al., 2022). Moreover, TC has displayed a positive influence on the cognitive function of PD patients. A meta-analysis reveals that TC can improve overall cognitive function in PD patients and has a trend toward promoting executive function (Yin et al., 2023). It is proposed to experience TC intervention twice a week, for 45–60 min each time, for at least 12 weeks to increase cognitive improvement. The most convincing evidence comes from a 3.5-year cohort study. This study displayed that in comparison to the control group who cannot exercise, PD patients who persisted in TC training had a significantly slower annual deterioration rate of the Unified PD Rating Scale, and the requirement for enhanced anti PD drug treatment was effectively decreased. Furthermore, the annual enhancement in equivalent daily dose of levodopa was also smaller (Li et al., 2024). This determining that TC not only promotes symptoms, but may also have a long-term neuroprotective effect in decreasing disease development.

### 3.4 Molecular mechanisms of TC

It is important to note that many of the molecular findings discussed herein, including alterations in BDNF, oxidative stress markers, and inflammatory cytokines, are primarily correlational in nature. While these changes are consistently associated with Tai Chi practice and correlate with improved functional outcomes, the direct causal links—demonstrating that Tai Chi *specifically and necessarily* induces these molecular changes which then *directly lead to* neural plasticity and clinical improvement—require further validation. Future mechanistic studies employing controlled component-disassembly designs, longitudinal biomarker assessments, and possibly animal models are needed to establish these causal chains.

#### 3.4.1 How TC influences neuroplasticity via neurotrophic factors, oxidative stress, and inflammatory pathways

**Figure 2:**
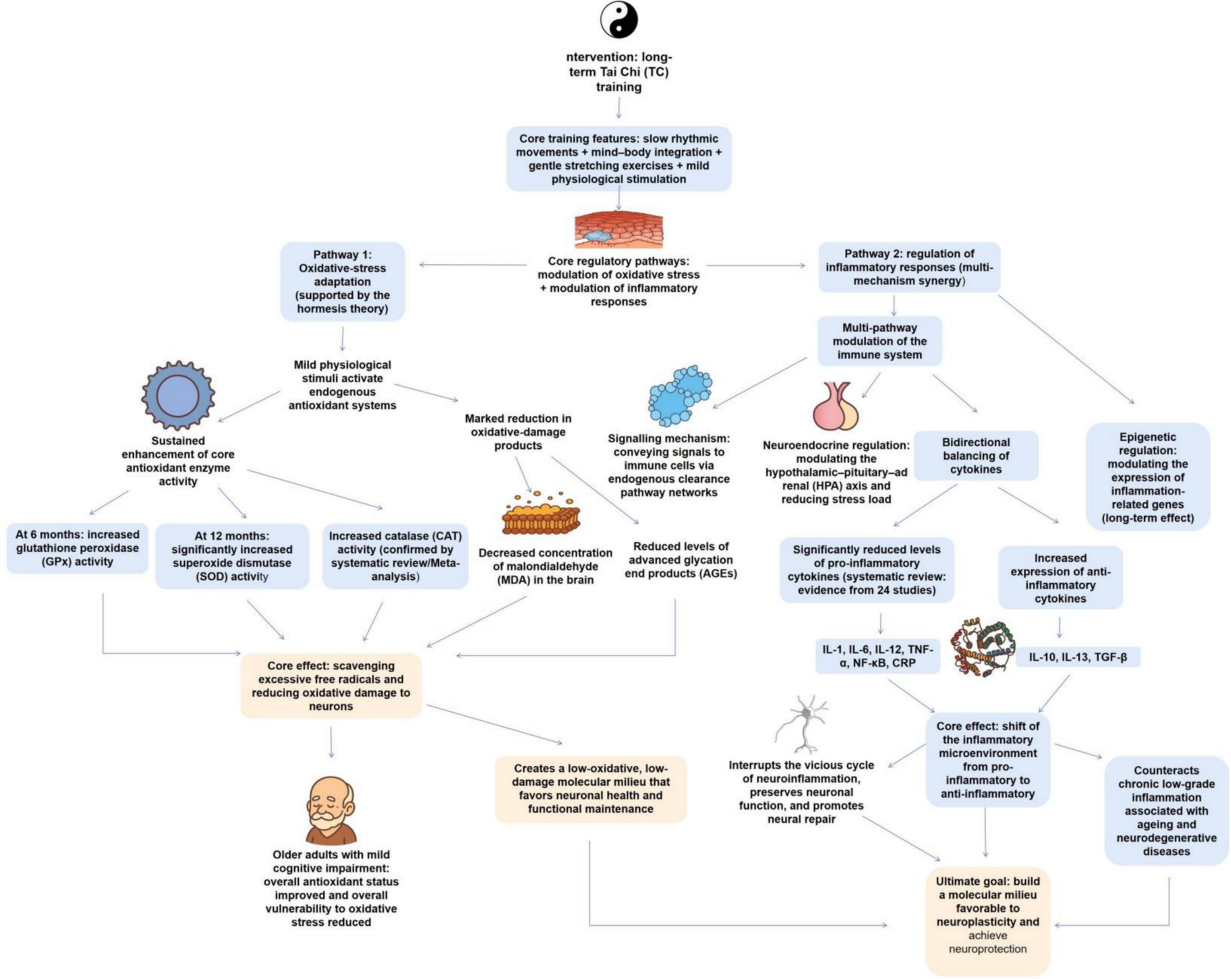
Molecular mechanism of TC promoting neuroplasticity through oxidative stress regulation and inflammation balance remodeling. This figure illustrates the two core molecular mechanisms by which long-term TC training affects neural plasticity: on the one hand, TC forms mild physiological stress through “slow paced rhythmic movements” and activates the endogenous antioxidant system based on the hormesis effect of toxins, continuously increasing the activity of core antioxidant enzymes such as SODGPx, oxidative damage products such as MDA and AGEs, and reducing neuronal oxidative damage, especially significantly improving the antioxidant status of elderly people with metabolic syndrome; On the other hand, TC synergistically regulates the immune system through multiple pathways such as mechanical conduction, neuroendocrine (HPA axis regulation), and epigenetics, and bidirectionally regulates cytokine balance - significantly reducing the levels of pro-inflammatory factors such as IL-1, IL-6, TNF - *α*, and increasing the expression of anti-inflammatory factors such as IL-10 and IL-13, shifting the inflammatory microenvironment from a pro-inflammatory state to an anti-inflammatory state, and blocking the vicious cycle of neuroinflammation. The above two pathways jointly create a molecular environment with low oxidative damage and low neuroinflammation, providing key support for neuronal health, repair, and neuroplasticity. The relevant conclusions have been validated by various types of evidence such as longitudinal studies, systematic reviews, and meta-analyses.

Neuroplasticity relies on the coordinated regulation of redox homeostasis, inflammatory balance, and neural nutrition. TC targets these three pathways to create a neuroprotective internal environment. Oxidative stress is a key trigger for neuronal damage, and TC triggers adaptive regulation through unique motor characteristics to strengthen endogenous antioxidant defense and alleviate oxidative damage from the source (Figure 2).

##### 3.4.1.1 Modulating oxidative stress: increasing endogenous antioxidant defenses

Oxidative stress is a vital pathological factor causing neuronal damage, accelerated brain aging, and the onset of neurodegenerative diseases (Kong et al., 2024). TC practice increases the body’s endogenous antioxidant defense capacity, effectively decreasing oxidative damage and conferring protection to the nervous system.

The mechanism of action initially manifests in the maintained upregulation of core antioxidant enzyme activities. A 12-month longitudinal study elucidated the dynamic impacts of TC exercise: at the initial intervention stage (6 months), practitioners exhibited a significant enhancement in glutathione peroxidase (GPx) activity; upon continuation to 12 months, superoxide dismutase (SOD) activity was also markedly elevated, concurrently with significant decreases in plasma concentrations of the lipid peroxidation product malondialdehyde (MDA) and advanced glycation end products (AGEs) (Goon et al., 2009). This mechanism along with the theory of hormesis, wherein TC, as a mild physiological stressor, may initially drive slight oxidative stress, hence activating and potentiating the body’s antioxidant defense system and ultimately obtaining long-term enhancement in redox status (Goon et al., 2009). This finding is robustly encouraged by a systematic review and meta-analysis, the findings of which determined that regular TC practice significantly increases the activities of superoxide dismutase (SOD) and catalase (CAT), while effectively decreasing levels of lipid peroxides in comparison to sedentary behavior (Rosado-Pérez et al., 2021). This displays that the impact of TC in augmenting antioxidant defense and decreasing oxidative damage is robust and reproducible.

Moreover, this systemic antioxidant effect is also significant in elderly populations with metabolic syndrome. Research determined that subjects experiencing 6 months of TC training exhibited a significantly enhanced total antioxidant status and a markedly decreased overall oxidative stress score (Mendoza-Núñez et al., 2018). Basic research offers a potential neurobiological association: the level of oxidative stress in the brain is tightly linked to memory function, and regulating redox balance represents a potential pathway for cognitive improvement (Kruk-Slomka et al., 2016).

Overall, via its slow, rhythmic movements, TC drives a mild physiological stress. This stress, far from being detrimental, initiates and improves the endogenous antioxidant system. By triggering the activities of core enzymes, such as SOD, CAT, and GPx, it effectively scavenges excess free radicals and mitigates oxidative damage, such as lipid peroxidation, ultimately establishing a molecular environment for the brain with decreased oxidative damage, one that is more conducive to neuronal health and functional maintenance.

##### 3.4.1.2 Modulating inflammatory responses: reshaping immune balance to offer neuroprotection

Chronic inflammation is a core pathological inducer in the onset and progression of different neurodegenerative diseases, such as Alzheimer’s disease (AD) and PD. A persistent inflammatory state disturbs neuronal function and impedes neural repair via the release of numerous pro-inflammatory factors (Li et al., 2021; Chitnis and Weiner, 2017). As a mind–body exercise, TC can regulate the immune system via numerous pathways, effectively decreasing systemic inflammation levels, hence establishing an anti-inflammatory microenvironment for the brain.

The anti-inflammatory impacts of TC are first evident in the precise modulation of core pro-inflammatory and anti-inflammatory cytokines. A systematic review synthesizing 24 studies uncovered that TC practice significantly decreases levels of different pro-inflammatory mediators, comprising interleukin-1 (IL-1), interleukin-6 (IL-6), interleukin-12 (IL-12), tumor necrosis factor-alpha (TNF-*α*), nuclear factor-kappa B (NF-κB), and C-reactive protein (CRP) (Liu et al., 2022). Concurrently, it elevates the expression of cytokines with anti-inflammatory properties, such as interleukin-10 (IL-10) and interleukin-13 (IL-13) (Liu et al., 2022). This bidirectional modulation of inflammatory balance is critical for controlling the vicious cycle of neuroinflammation. The potential process involves complex, multi-level regulation. Firstly, the unique slow stretching movements of TC are hypothesized to transmit mechanical signals directly to immune cells via the fascial network, inhibiting their generation of pro-inflammatory factors (e.g., IL-6, TNF-α) and facilitating the expression of anti-inflammatory factors (e.g., IL-10, TGF-*β*) (Thelander and Ring, 2025). Secondly, the mind–body combination characteristic of TC enables it to regulate the function of the hypothalamic–pituitary–adrenal (HPA) axis, decreasing chronic stress responses, which are a significant driver of inflammation (Thelander and Ring, 2025). Furthermore, TC may exert long-term immunomodulatory impacts via epigenetic pathways, modulating the expression of inflammation-associated genes (Thelander and Ring, 2025). This alter from a pro-inflammatory toward an anti-inflammatory state holds significant immunological significance. As uncovered in chronic inflammatory diseases, such as rheumatoid arthritis, restoring immune balance and facilitating the resolution of inflammation are core to treatment (Chen et al., 2019). TC obtains this by driving a similar, balance-oriented immune phenotype, counteracting the chronic low-grade inflammation linked to aging and neurodegenerative diseases (Irwin and Olmstead, 2012; Liu et al., 2021).

Overall, TC, via mechanotransduction, neuroendocrine regulation, and potential epigenetic mechanisms, acts synergistically on the immune system to alter the inflammatory milieu from a destructive pro-inflammatory state toward a protective anti-inflammatory state. This systemic immunomodulatory action effectively attenuates neuroinflammation in the brain, offers protection for neurons, and establishes a molecular environment favorable for neuroplasticity and repair.

#### 3.4.2 Potential mechanisms of TC in neuroprotection and cellular metabolism

**Figure 3:**
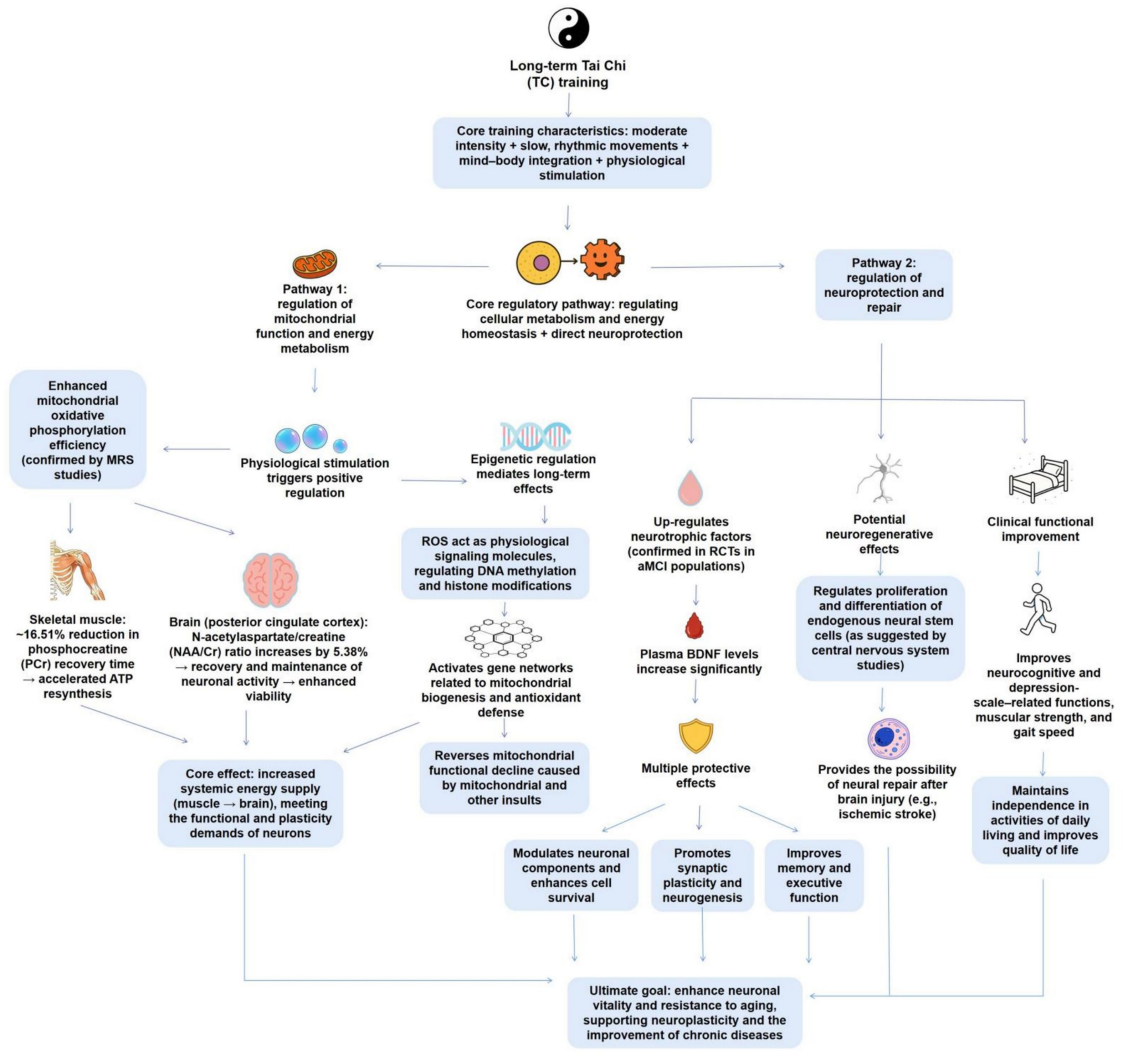
The mechanism by which TC promotes neuroplasticity by regulating cellular metabolism and neuroprotection. This figure presents the core mechanism of long-term TC training in neuroprotection and cellular metabolism: TC training characterized by “moderate intensity+slow paced movements” works synergistically through two pathways. On the one hand, TC, as a physiological stimulus, directly enhances mitochondrial oxidative phosphorylation efficiency, shortens PCr recovery time, and increases the NNAA/Cr ratio in brain regions. At the same time, it activates mitochondrial biogenesis related genes through ROS mediated epigenetic regulation (DNA methylation/histone modification), achieving optimized whole-body energy supply from muscle to brain; On the other hand, TC practice is associated with elevated levels of BDNF, a neurotrophic factor implicated in neuronal survival and synaptic plasticity. This suggests that BDNF upregulation may be one of the molecular pathways through which TC confers neuroprotective effect, providing support for brain injury repair and improving clinical function in patients with neurocognitive disorders.

The neuroprotective impacts of TC are mainly manifested via its modulation of cellular metabolism and energy homeostasis. By regulating mitochondrial function and promoting cellular metabolism, TC contributes to increased viability and resilience of neural cells (Figure 3).

##### 3.4.2.1 Mitochondrial function and energy metabolism

Mitochondria, acting as the cellular powerhouses, play an indispensable role in experiencing the high energy demands of neurons, sustaining calcium homeostasis, and determining cell survival (Walters and Usachev, 2023; Zhang et al., 2025). Their dysfunction is intimately linked to the pathological mechanisms of aging and different neurodegenerative diseases (Walters and Usachev, 2023; Zhang et al., 2025). Literature has determined that TC practice can act as an positive physiological stimulus, encouraging normal neuronal function and increasing resilience against stress and injury by improving mitochondrial function and hence augmenting energy supply to neurons (Zhou et al., 2018).

Its positive influence is first evident in the direct improvement of energy metabolic effectiveness. A pioneering magnetic resonance spectroscopy (MRS) study offered initial evidence: after 12 weeks of TC training, elderly subjects exhibited a significant 16.51% decrease in phosphocreatine (PCr) recovery time in their leg muscles (Zhou et al., 2018). The PCr recovery time is a core indicator of mitochondrial oxidative phosphorylation capacity and ATP resynthesize rate; its shortening clearly reveals that TC training increases mitochondrial energy metabolic effectiveness, enabling cells to replenish their energy reserves more widely after expenditure (Zhou et al., 2018).

The molecular processes potential this improvement of mitochondrial function may involve epigenetic regulation. Reactive oxygen species (ROS) generated by regular exercise are no longer viewed only as harmful byproducts; at physiological levels, they act as critical signaling molecules. Via the modulation of epigenetic mechanisms, such as DNA methylation and histone modifications, they activate gene expression networks linked to mitochondrial biogenesis and antioxidant defense, hence reversing mitochondrial functional decline driven by factors, such as aging (Li et al., 2022). TC, as a moderate-intensity exercise, similarly sustains the health and stability of these cellular power plants by driving this advantageous “epigenetic-mitochondrial” crosstalk (Li et al., 2022). It is worth noting that these energetic benefits similarly extend directly to the brain. The same MRS study also uncovered a significant 5.38% enhancement in the ratio of N-acetylaspartate to creatine (NAA/Cr) in the posterior cingulate cortex of elderly subjects following TC practice (Zhou et al., 2018). NAA is a marker for neuronal mitochondrial integrity and neuronal viability; its elevated level strongly reveals that TC training improves energy metabolism and health status within brain neurons (Moffett et al., 2007). This enrouarges the model of communication between the nervous system and peripheral tissues via “mitochondrial stress signals,” whereby physical interventions, such as TC can produce systemic impacts and ultimately influence brain health and overall organismal aging by regulating core biological mechanisms, such as mitochondrial function (Li et al., 2022).

Overall, TC practice systemically increases cellular energy supply levels from muscle to the brain by promoting mitochondrial energy metabolic effectiveness, potentially consolidating this impact via epigenetic processes. This not only offers more abundant “fuel” for nerve cells to meet daily functional demands and different stresses, but also lays a solid energetic foundation for maintaining long-term neuroplasticity and healthy aging.

##### 3.4.2.2 Neuroprotective effects: inhibiting apoptosis and facilitating neural repair

Beyond its preventive advantages, TC also exerts significant neuroprotective impacts by directly decreasing nerve cell death and actively facilitating neural repair (Sungkarat et al., 2018; Lin et al., 2024). This protective effect, induced via the modulation of core intracellular signaling pathways, ultimately helps in repairing damaged neural networks and promoting neurological function (Sungkarat et al., 2018; Lin et al., 2024).

The neuroprotective role of TC is first displayed by its capacity to counteract apoptosis and promote neuronal survival. A RCT involving older adults with amnestic Mild Cognitive Impairment (aMCI) offered direct evidence. The study uncovered that subjects who experienced 6 months of TC training exhibited significantly higher plasma levels of BDNF in comparison to the control group receiving health education (Sungkarat et al., 2018). BDNF is a core factor maintaining neuronal survival, facilitating synaptic plasticity, and encouraging neurogenesis. The upregulation of its level represents a core molecular basis for the neuroprotective impacts of TC, tightly linking to the uncovered enhancements in memory and executive function in the study (Sungkarat et al., 2018). Moreover, multi-component training incorporating TC elements has also been displayed to effectively promote functional fitness in patients with neurocognitive disorders, such as lower limb mobility and walking speed, which is critical for maintaining their independence in daily living and quality of life, indirectly implying its protective advantages for neurological function (Ribeiro et al., 2021).

It is worth noting that the neuroprotective mechanisms of TC may expand to facilitating endogenous neuroregeneration. Research determining that under pathological conditions, such as ischemic stroke, improving the proliferation and differentiation of endogenous neural stem cells to replace damaged nerve cells is a significant future direction for brain repair therapies (Tang et al., 2023). Although more direct evidence is required, therapies within the traditional Chinese medicine system, of which TC is a significant mind–body practice, have been uncovered to specifically regulate the proliferation and differentiation of neural stem cells by regulating numerous signaling pathways and related transcription factors (Qin et al., 2017). This strongly reveals that TC, as a traditional mind–body exercise, may possess similar potential to promote neurogenesis after brain injury by activating endogenous repair processes.

Overall, TC inhibits apoptosis and increases neuronal resilience by upregulating levels of neurotrophic factors, such as BDNF, and may promote neural regeneration and repair by regulating the activity of endogenous neural stem cells. These processes collectively constitute the multi-level neuroprotective strategy of TC, offering a theoretical basis for its potential effectiveness in promoting neurological function and repairing damaged networks under pathological conditions, such as stroke and brain injury.

### 3.5 The utilization of TC in neurological rehabilitation

#### 3.5.1 Utilization examples of TC in neurological rehabilitation: rehabilitation of stroke and brain injury patients

TC, as a low-intensity aerobic exercise, has been extensively utilized in the range of neurological rehabilitation, notably in the rehabilitation mechanism of stroke and brain injury patients. Stroke and brain injury commonly cause impairments in motor function, balance, cognitive function, and daily living abilities. TC training can effectively promote the motor function and quality of life of these patients (Table 2).

**Table 2:** Main research achievements on the core effects and key mechanisms of TC in the field of neural rehabilitation applications.

Application field	Core effects	Key mechanisms	Main research findings	References
Neurological rehabilitation—stroke patients	1. Improves motor function, balance ability, and reduces fall risk; 2. Promotes upper and lower limb function recovery; 3. Enhances cardiovascular function, emotional state, and cognitive function; 4. Improves activities of daily living (ADL) and quality of life	1. Low-intensity aerobic exercise characteristics; 2. Enhances limb coordination and muscle strength; 3. Integrates physical and mental regulation effects	1. Significantly increases grip strength, 6-min walking distance, and eyes-open single-leg standing time; 2. Seated TC improves upper limb function and sitting balance, and alleviates depressive symptoms; 3. Superior to conventional rehabilitation in improving Berg Balance Scale and Fugl-Meyer motor scores	Wehner et al. (2021), Chen et al. (2023), Zhang et al. (2023), Zhao et al. (2022), Tan et al. (2021) and Song et al. (2021)
Neurological rehabilitation—brain injury patients (including TBI)	1. Promotes improvements in cognitive, functional, and psychological outcomes; 2. Enhances emotional regulation ability; 3. Ensures safety and tolerability of rehabilitation	1. Increases clustering coefficient and local efficiency of brain functional networks, promoting neural plasticity; 2. Improves working memory capacity; 3. Low-impact movement characteristics	1. Consistently improves patients’ functional, psychological, and cognitive indicators; 2. Reduces fluctuations in emotional responses and enhances emotional stability; 3. No significant adverse events, with good safety	Solloway et al. (2016), Cui et al. (2021), Laskosky et al. (2024), Wang et al. (2023), Yang et al. (2022) and Yang et al. (2022)
Cognitive enhancement	1. Improves attention, memory, and executive function; 2. Delays age-related cognitive decline (e.g., MCI, early dementia)	1. Strengthens functional connectivity between the prefrontal cortex and hippocampus; 2. Enhances small-world properties of brain networks; 3. Facilitates neuroadaptive changes	1. Significant improvement in memory quotient after 12 weeks of practice; 2. Enhances hippocampal-medial prefrontal cortex functional connectivity; 3. Activates oxyhemoglobin concentration in bilateral prefrontal regions	Qi et al. (2023), Tao et al. (2016), Chen et al. (2022), Lim et al. (2019), Jasim et al. (2023), Li et al. (2024) and Boa Sorte Silva et al. (2024)
Chronic pain management	1. Relieves chronic pain (e.g., osteoarthritis, low back pain, fibromyalgia); 2. Improves physical function and quality of life	1. Enhances muscle strength, joint flexibility, and postural control; 2. Reduces pro-inflammatory cytokine levels and regulates endocannabinoids; 3. Balances autonomic nervous function and reduces stress responses	1. Significantly decreases pro-inflammatory factors such as IL-6 and TNF-α; 2. Improves heart rate variability and reduces perceived stress; 3. Has immediate analgesic effects on osteoarthritis and low back pain	Wehner et al. (2021), Zou et al. (2018), Liu et al. (2022), Shen et al. (2023), Kong et al. (2016) and Peng (2012)

##### 3.5.1.1 Stroke patients

The most significant advantages of TC for stroke patients are implied in its improvement of motor function, balance ability, and fall risk. A systematic review and meta-analyses have determined that TC training can significantly promote different physical fitness indicators, comprising grip strength, 6 min walking distance, and single leg standing time with eyes open (Wehner et al., 2021). Another large-scale meta-analysis targeting the elderly further determined that TC can effectively decrease the risk of falls and promote balance indicators, such as timed standing and walking tests and functional extension tests (Chen et al., 2023). These advantages are critical for stroke patients with hindered balance function and high risk of falls. Long term practice of TC can continuously promote the motor function of stroke patients and accelerate the recovery of their upper and lower limb functions. A systematic review and meta-analysis specifically targeting the impacts of TC Yunshou on stroke patients displayed that in comparison to conventional rehabilitation training, TC Yunshou training significantly promoted patients’ Berg Balance Scale scores, simplified upper limb function test scores, and displayed greater enhancement in Fugl Meyer exercise evaluation (Zhang et al., 2023). This determining that TC has a unique effect in facilitating movement management and coordination.

For stroke patients in the subacute phase with limited mobility, the promoted sitting style TC is equally positive. A rigorous RCT displayed that a 12 week sitting style TC training can significantly promote upper limb function (assessed by Wolf Motor Function Test), balance control, sitting balance, and decrease depressive symptoms in patients (Zhao et al., 2022). This offers feasible rehabilitation plans for patients with various degrees of functional impairment. Moreover, the advantages of TC go far beyond the range of exercise function, expanding to the improvement of cardiovascular function, emotional stability, and cognitive function, integratively facilitating patients’ return to daily life. A research plan seeks to investigate the impacts of TC on cardiorespiratory adaptation in stroke patients during the recovery period, with a center on evaluating peak oxygen uptake and quality of life, determining the possible role of TC in promoting overall physiological function (Tan et al., 2021). Another randomized feasibility study uncovered that rehabilitation programs according to TC were superior to conventional symptom control programs in promoting flexor muscle strength, walking ability, daily living activity ability, and cognitive function, and displayed significant benefits in the thinking and self-care dimensions of stroke particular quality of life scales (Song et al., 2021).

Overall, TC offers a integrative rehabilitation pathway for stroke patients via its characteristic of both physical and mental cultivation. It can not only effectively increase limb coordination, muscle strength and balance, decrease the risk of falls, but also accelerate the recovery of motor function, and simultaneously promote the patient’s cardiovascular health, emotional state and cognitive ability, ultimately significantly promoting their daily living activities and overall quality of life, aiding patients better reintegrate into society.

##### 3.5.1.2 Patients with brain injury

For patients with brain injury, notably those with traumatic brain injury (TBI), TC, as a low impact physical and mental exercise, exhibits unique therapeutic value in the rehabilitation process. Although high-quality research directly targeting brain injury patients is still in the accumulation stage, recent systematic evaluations show positive signals. A systematic review specifically evaluating the effectiveness of TC and Qigong in treating traumatic brain injury uncovered that TC consistently promoted patients’ functional, psychological, and/or cognitive outcomes in several included trials (Laskosky et al., 2024). The review indicates that according to these consistent positive findings, it is sufficient to encourage large-scale, high-quality multicenter trials to further validate the effectiveness of TC in traumatic brain injury, determining that TC may turned into a new frontier in the range of brain injury treatment (Laskosky et al., 2024).

The rehabilitation mechanism of TC on patients with brain injury may stem from its positive influence on brain function and structure. Although most neuroimaging study are conducted in healthy populations, the patterns they suggest have enlightening implications for determining brain injury rehabilitation. Research has uncovered that TC training can significantly increase the clustering coefficient and local effectiveness of brain functional networks, facilitating brain functional specialization (Cui et al., 2021). This enhancement in neural plasticity is notably implied in brain regions linked to cognitive processing, such as the thalamus, and has been uncovered to be a predictor of promoted cognitive flexibility. For brain injury patients whose neural network connections are commonly disrupted, TC’s ability to promote brain function reorganization and improvement may be the neural basis for promoting cognitive functions, such as executive function and working memory. Meanwhile, TC has displayed clear benefits in promoting emotional regulation. Research has displayed that practicing TC can significantly decrease valence fluctuations, arousal fluctuations, and dominance fluctuations in emotional responses, determining that it can effectively increase emotional stability (Wang et al., 2023). It is interesting that researchers speculate that the action memory training in TC may increase emotional regulation ability by promoting working memory capacity. This has significant psychological rehabilitation importance for brain injury patients who commonly have emotional instability, difficulty in impulse control, and depressive symptoms.

It is worth noting that the low impact nature of TC makes it a safe and well tolerated rehabilitation option for patients with brain injuries. Systematic assessments have displayed that TC involves several slow, low impact movements that combine breathing, thought, and physical activity, and has good safety (Solloway et al., 2016). A systematic review of traumatic brain injury also indicated that studies displaying adverse events displayed that neither the TC group nor the control group experienced adverse events, preliminarily determining its safety (Laskosky et al., 2024). Moreover, the role of TC in promoting emotional states, decreasing anxiety and depression symptoms has been extensively determined. The system evaluation displays that TC can significantly decrease anxiety and depression levels, and promote the psychological health dimension of quality of life (Yang et al., 2022). This is critical for patients with brain injuries to rebuild their confidence and quality of life. A large-scale systematic review reported that TC has multidimensional advantages, comprising physical, psychological, and quality of life perspectives, and is applicable to different health conditions (Yang et al., 2022). This conclusion also encourages its potential utilization in integrative rehabilitation of brain injuries.

Overall, preliminary evidence reveals that TC can assist cognitive recovery by facilitating brain functional plasticity and promote psychological state by increasing emotional regulation ability for patients with brain injuries. Its low impact features ensure the safety of the rehabilitation process. Via systematic TC training, patients can not only recover in motor function and developed cognitive function, but also receive strong encourage in psychological and emotional perspectives, hence obtaining integrative physical and mental recovery and effectively promoting their quality of life.

#### 3.5.2 The possible role of TC in cognitive enhancement and chronic pain control

Beyond its rehabilitative impacts on motor function, TC displays potential in cognitive enhancement and chronic pain management, notably for elderly populations and individuals suffering from chronic pain conditions (Table x).

##### 3.5.2.1 Cognitive enhancement

The advantageous impacts of TC on cognitive function mainly stem from its positive remodeling of core neural structures and functional networks in the brain (Tao et al., 2016; Chen et al., 2022). Research determining that TC effectively increases different cognitive domains—including attention, memory, and executive function—by specifically augmenting activity and connectivity within brain regions critically involved in higher-order cognitive processing, notably the prefrontal cortex and hippocampus (Tao et al., 2016; Chen et al., 2022).

The neural processes potential these cognitive benefits have been visually elucidated via functional brain imaging studies. A longitudinal study involving older adults uncovered that after 12 weeks of TC or Baduanjin practice, participants displayed a significant enhancement in memory quotient (Tao et al., 2016). It is worth noting that resting-state functional magnetic resonance imaging (fMRI) suggested significantly enhanced functional connectivity between the bilateral hippocampus and the medial prefrontal cortex, and this enhancement in connectivity was effectively linked to the degree of memory improvement (Tao et al., 2016). This finding strongly reveals that TC may counteract age-associated memory decline by improving the functional combination of this core hippocampo-prefrontal memory circuit (Tao et al., 2016). In addition to improving memory circuits, TC also exerts a significant activating impact on the prefrontal cortex itself, which is responsible for executive control. A study using functional near-infrared spectroscopy (fNIRS) uncovered brain activity during TC Zhan Zhuang (a core static meditative practice). The findings displayed that during Zhan Zhuang, practitioners exhibited a significant enhancement in oxyhemoglobin concentration in bilateral prefrontal regions, comprising the dorsolateral and ventrolateral prefrontal cortex, alongside increased functional connectivity between the left and right prefrontal cortices. The brain’s functional network also displayed more efficient small-world properties (Qi et al., 2023). This proves that TC practice directly increases neural activity within the prefrontal cortex and improves its functional organization, offering a direct physiological basis for the enhancements in executive function, working memory, and complex problem-solving abilities linked to TC.

In older populations, the translation of these neural processes manifests as a decrease in cognitive decline, notably the progression toward dementia, such as AD. A systematic literature review noted that for older adults in the early stages of dementia, TC displays possible role for promoting global cognition, visuospatial skills, semantic memory, and verbal learning/memory (Lim et al., 2019). Nevertheless, a scoping review also indicates that recent findings concerning the neurocognitive advantages of TC for individuals with Mild Cognitive Impairment (MCI) and early dementia are inconsistent, necessitating more high-quality clinical trials and mechanistic studies to explain its effectiveness and pathways of action (Jasim et al., 2023). The potential reason for TC’s improvement of brain health lies in its unique integration of aerobic exercise and complex coordinative training. This combined mind–body movement pattern aids sustain an active brain state. Study reveal that physical exercise can improve brain plasticity via mechanisms, such as the modulation of neural oscillations (Li et al., 2024). Overall, exercise training is a critical approach for counteracting the decline in cognition and brain health during aging, with its advantages similarly induced by driving neuroadaptive modifications (Boa Sorte Silva et al., 2024).

Overall, TC encourages and increases cognitive function across numerous levels by enhancing functional activity and connectivity within the prefrontal cortex and hippocampus, and possibly by regulating brain network effectiveness and plasticity. As a mind–body exercise that incorporates both aerobic and coordinative demands, it displays a promising non-pharmacological intervention approach for decreasing age-associated cognitive decline and neurodegeneration.

##### 3.5.2.2 Chronic pain control

Similar to yoga and mindfulness-based stress reduction, TC demonstrates efficacy in managing chronic pain conditions like osteoarthritis and low back pain, likely through shared mechanisms including improved physical function, downregulation of systemic inflammation, and enhanced pain coping psychology. The slow, gentle, and continuous movement patterns of TC offer a direct physical basis for its pain-relieving effects. A systematic review and meta-analysis determined that TC training significantly promotes different physical fitness parameters, comprising grip strength, 6-min walk distance, single-leg stance time, and thoracolumbar flexibility (Wehner et al., 2021). By increasing muscle strength, promoting joint flexibility, and improving postural control, TC can effectively correct biomechanical abnormalities caused by negative posture and muscular imbalances, hence decreasing mechanical pain in the musculoskeletal system at its source.

Beyond physical enhancements, the analgesic impact of TC is more significantly implied in its modulation of the biochemical environment linked to pain. A pilot study involving postmenopausal women with knee osteoarthritis suggested the mechanism of TC: after 8 weeks of practice, participants displayed significantly decreased levels of pro-inflammatory oxylipins (e.g., PGE1 and PGE2) in their circulation, alongside corresponding modifications in the levels of endocannabinoids, which are tightly linked to pain perception and neuroinflammation (Shen et al., 2023). These modifications in biochemical markers linked to patients’ pain evaluations and modifications in functional connectivity between the amygdala and prefrontal cortex, implying that TC may exert integrated anti-inflammatory and analgesic impacts both peripherally and centrally by regulating the neuro-immune axis (Shen et al., 2023). Systematic reviews encourage this view, determining that TC practice can decrease levels of different pro-inflammatory cytokines, comprising interleukin-6, tumor necrosis factor-alpha, and C-reactive protein (Liu et al., 2022).

Moreover, the relaxation and meditative components inherent in TC play a core role in decreasing pain triggered by psychological stress. A meta-analysis of mind–body exercises (TC/Yoga) displayed that these practices significantly promote heart rate variability parameters, displayed by reduced low-frequency power and enhanced high-frequency power, while effectively decreasing perceived stress levels (Zou et al., 2018). This determining that TC can decrease stress responses by increasing parasympathetic tone and regulating the sympathetic-vagal balance, hence indirectly decreasing chronic pain conditions tightly related to stress (Zou et al., 2018).

Substantial clinical research evidence enrouarges the utilization of TC for particular chronic pain conditions. A meta-analysis incorporating 18 RCTs displayed that TC has significant immediate impacts on relieving chronic pain triggered by osteoarthritis, low back pain, and osteoporosis (Kong et al., 2016). Review articles further affirm that TC, as an ancient health art combining adapted movement, mind–body interaction, and meditation, displays clear effectiveness in controlling conditions, such as osteoarthritis, fibromyalgia, and low back pain (Peng, 2012).

Overall, the decrease of chronic pain by TC findings from the synergistic action of numerous factors. It relies not only on physical effects, such as promoted physical function and improved blood circulation but, more vitally, exerts integrative impacts across numerous stages of pain—comprising perception, transmission, and regulation—by regulating the nervous system (e.g., autonomic balance) and the immune system (e.g., decreasing systemic inflammation). Hence, TC can be considered an positive complementary and alternative therapy, suitable for combination into integrative control approaches for chronic pain. Table 2 shows Main research achievements on the core effects and key mechanisms of TC in the field of neural rehabilitation applications.

### 3.6 Limitations and future directions

#### 3.6.1 Limitations of existing evidence

There are several common methodological limitations in current research that may affect the robustness and universality of the conclusions. There are significant differences in the forms, intensity, frequency, duration, and total cycle of Tai Chi intervention in literature, such as Yang’s, Chen’s, and simplified 24 forms. For example, some studies use simplified 24 style Tai Chi twice a week for 60 min each time, while others use more complex traditional routines or combine standing exercises. This inconsistency in “dosage,” coupled with the diversity of control group settings (from routine care, health education to other types of exercise), has led to high heterogeneity among research results, requiring careful interpretation of cross study comparisons and meta-analysis conclusions. The lack of standardized and reproducible intervention protocol reporting guidelines for specific populations, such as Parkinson’s disease patients and mild cognitive impairment patients, is a core obstacle to evidence integration and clinical promotion. Firstly, achieving complete double-blind intervention in sports is almost impossible, which may lead to expected effects or performance biases. Although the use of “attention control groups” (such as low-intensity stretching and health education courses) can partially alleviate this problem, many early studies only used “routine care” controls, weakening the strength of causal inference. Secondly, some studies have small sample sizes and insufficient statistical power, which may overlook subtle but important effects or lead to false positive results. Furthermore, the follow-up period of most studies is relatively short (usually 3–6 months), and we know very little about the long-term sustainability of the benefits of Tai Chi and the persistent form of its “dose–response” relationship. Although neuroimaging shows that changes such as increased hippocampal volume and upregulation of BDNF levels are associated with cognitive improvement as described in this article, these findings are still fundamentally correlated. We still lack direct evidence to prove that specific components of Tai Chi (such as slow movements, breathing regulation, meditation focus) are necessary conditions for triggering these neurobiological changes. Most molecular level studies are based on peripheral blood biomarkers such as plasma BDNF and inflammatory factors, and their direct relationship with actual changes in the central nervous system still needs further validation.

A primary limitation across the field is the predominantly correlational nature of the evidence linking Tai Chi to neurobiological changes. Most studies demonstrate temporal association but cannot rule out confounding factors or establish definitive causality. Proving mechanistic causality remains a key challenge. Future research must move beyond association by: (1) designing studies that can test the necessity of specific Tai Chi components; (2) utilizing experimental paradigms (e.g., mediation analysis with multiple timepoints) that better support causal inference; and (3) integrating measures that capture temporal dynamics between practice, molecular changes, and neural/behavioral outcomes. Beyond the heterogeneity of interventions, many studies, particularly those employing costly neuroimaging or detailed molecular assays (e.g., Cha et al., 2024), are limited by small sample sizes. This reduces statistical power, increases the risk of both Type I and Type II errors, and limits the generalizability of findings.

#### 3.6.2 Inconsistent research findings and exploration of controversial areas

Objectively presenting inconsistent evidence is the fundamental responsibility of scientific reviews. As mentioned earlier, the cognitive benefits of Tai Chi for MCI patients are not entirely consistent. Some high-quality studies have found significant improvements in overall cognition or specific memory domains, while others have reported insignificant effects or are only effective for certain subgroups. This inconsistency may stem from: (1) heterogeneity in the MCI population: amnestic and non-amnestic MCI may have different responses to interventions; (2) The difference between intervention plan and control group: Different “exercise doses” and the level of control activity directly affect the comparison of effect size; (3) Sensitivity of outcome measurement: Some cognitive tests may not be able to capture subtle, specific cognitive changes brought about by Tai Chi (such as executive functions related to action planning). Future research needs to stratify MCI more finely and use cognitive assessment tools that are more sensitive to physical and mental interactions It is necessary to acknowledge and discuss studies that have not found significant effects of Tai Chi on specific outcomes, such as inflammation markers or functional connections in specific brain regions. These “negative” or “invalid” results are crucial for a comprehensive understanding of the boundary conditions of the intervention. At present, there may be some publication bias in this field, where positive results are more likely to be published, which may lead to an overestimation of the effects of Tai Chi. Encourage prospective registration of future research and publication of all results, regardless of their statistical significance.

#### 3.6.3 Key directions and challenges for future research

To overcome the above limitations and deepen the field, we propose the following priority research directions Component disassembly research: Design experiments to separate or recombine the “body movements,” “breathing regulation,” and “mental focus” components of Tai Chi to identify their unique contributions to neural plasticity. Construct a multidimensional mechanism network in the human body by combining neuroimaging, epigenetics, metabolomics, and proteomics. At the same time, develop animal models that simulate the core features of Tai Chi to explore more controllable causal mechanisms and validate targets. Utilizing emerging technologies to more directly explore the real-time correlation between changes in peripheral biomarkers and alterations in brain structure/function. Consensus needs to be reached on the “core” intervention plans for different target populations (such as PD, post-stroke, MCI) in the field, and detailed reporting on their elements (style, intensity, frequency, duration) should be provided. On this basis, explore the “precision health” pathway: by collecting baseline genotypes (such as BDNF Val66Met polymorphism), neuroimaging features, clinical phenotypes, and biomarkers, predict individual responses to Tai Chi intervention, and develop personalized prescriptions. The research perspective should shift from short-term efficacy to long-term benefits. More rigorous long-term follow-up studies (≥ 2 years) are needed to evaluate the potential impact of Tai Chi on the progression of neurodegenerative diseases (such as whether it can truly delay the transition from MCI to dementia). In addition, how to integrate effective Tai Chi intervention programs into routine healthcare systems and community projects, and how to use digital technologies (such as video courses, wearable devices, mobile applications) to increase participation, ensure movement compliance, and achieve remote guidance, are key scientific issues related to the implementation of its public health impact.

## 4 Conclusion and outlook

### 4.1 Conclusion

In contrast to earlier narrative summaries, this review provides an integrative analysis that not only consolidates the positive effects of TC but also, through a critical lens, examines the consistency of evidence, discusses common methodological pitfalls (e.g., small sample sizes, heterogeneity), and explicitly contrasts TC with related mind–body interventions to delineate its potential unique and shared mechanisms. Our review combines multi-level evidence from molecules to systems, from structure to function, clearly displaying that TC is not a simple physical activity, but a powerful mind body intervention that effectively induces brain neural plasticity via multi process synergy. Its core mechanism of action stems from its unique physical and mental combination characteristics. The slow, smooth, and complex sequence of movements constitutes a continuous sensory movement and cognitive challenge, while deep breathing and mental focus introduce elements of meditation and mindfulness. These two work together to modulate the autonomic nervous system, increase parasympathetic nervous activity, and create an improved physiological environment for neural plasticity. At the level of neural structure, long-term TC practice can effectively combat age-associated brain atrophy, notably by enhancing the gray matter volume and density in the hippocampus and prefrontal cortex, which are tightly linked to memory and executive function. Functionally, neuroimaging evidence suggests that TC drives functional specialization and effectiveness improvement of brain networks, manifested as a “streamlining” of internal connections in core networks, such as executive control networks and default mode networks, implying higher neural processing effectiveness and linked to better cognitive performance and emotional regulation abilities. At the molecular level, TC directly encourages neuronal survival and synaptic plasticity by upregulating core neurotrophic factors, such as BDNF. Meanwhile, it offers a strong protective microenvironment for neurons by increasing the endogenous antioxidant defense system and shifting the immune balance toward an anti-inflammatory state. Moreover, TC can improve mitochondrial energy metabolism, determining that nerve cells have sufficient energy supply. These processes ultimately translate into broad clinical and health benefits, including, but not limited to: significant enhancements in motor function and balance, and decreased risk of falls; Increase cognitive abilities, such as memory and executive function; effectively modulate emotions, decrease anxiety and depression symptoms; And play a positive role in the rehabilitation of neurological diseases, such as PD and stroke, even showing the potential to decrease disease development. Overall, recent evidence strongly encourages viewing TC as a non-pharmacological, holistic brain health improvement and neurological rehabilitation approach according to neuroscience principles.

### 4.2 Utilization prospects of TC in clinical practice and rehabilitation

Based on a solid evidence foundation, the utilization prospects of TC in clinical and rehabilitation medicine are very extensive, and its value lies in its safety, accessibility, low cost, and high benefits. The large-scale improvement of TC in communities and elderly populations can function as a low-cost and positive public health intervention to avoid cognitive decline, sustain physical function, improve psychological wellbeing, and decrease the risk of falls. As a control and adjuvant therapy for neurodegenerative diseases, TC should be considered a significant component of the standard rehabilitation plan for PD patients. Evidence displays that it significantly promotes posture stability and gait, even surpassing conventional exercise. The future aspect is to investigate its synergistic effect with drug therapy and its possible role for disease modification. For MCI and early-stage dementia patients, TC is a highly promising non pharmacological cognitive intervention. Future research requires to explain its advantages for particular cognitive domains, such as memory and executive function, and determine the subgroups most similarly to respond. TC, comprising promoted sitting TC, is suitable for patients at various stages of rehabilitation after stroke. It can not only promote the recovery of motor function, but also synchronously solve general cognitive disorders and emotional problems, obtaining integrative rehabilitation. Efforts should be made to combine TC into the standard clinical pathway for stroke rehabilitation. The advantages of TC for chronic pain (such as osteoarthritis), cardiovascular disease, and metabolic syndrome are partly due to its regulatory impacts on the nervous system, stress response, and inflammation. This makes it an ideal supplement to integrative control plans for different chronic diseases. Future utilizations require to go beyond the “one size fits all” model. By integrating biomarkers, such as BDNF levels, genotypes, neuroimaging features, and clinical phenotypes, it may be possible to obtain “precise TC” in the future, tailoring the most positive exercise types, intensities, and durations for various individuals.

### 4.3 Core issues and challenges in future TC research

Although abundant evidence, the following core issues and challenges urgently require to be handled in order to further combine TC into the modern medical system. Most molecular and systemic process studies are still at the level of association, lacking direct causal evidence. It is necessary to integrate intervention research with multi omics analysis (such as proteomics, metabolomics), and utilize animal models to improve the core components of TC, in order to directly verify the necessity of particular molecular pathways in inducing its neuroprotective effects. There are many schools of TC (such as the Yang and Chen schools) with different forms (such as simplified 24 forms and TC standing stakes), and the intervention duration, frequency, and period are not consistent, causing high heterogeneity among studies and making it difficult to conduct reliable comparisons and meta-analyses. Consensus requires to be obtained in the range on the “optimal dosage” (including style, duration, frequency) for various populations and diseases, and standardized intervention plan displaying guidelines should be developed. It is extremely difficult to achieve double-blind intervention in sports, which can easily cause expected impacts and performance deviations. Attention control groups (such as low-intensity stretching and health education) should be utilized rather than just a “routine care” control group to better isolate the particular impacts of TC. For the findings assessed by. It is interesting that researchers, the evaluator blinding approach should be implemented. There are differences in individual responses to TC training, but recently little is determined about the factors that impact response, such as genes, baseline function, and personality traits. Future RCTs should comprise subgroup analysis and responder analysis to investigate biomarkers and clinical features that can predict treatment efficacy, offering a basis for individualized recommendations. Most studies have short follow-up periods and little is determined about the long-term sustainability of the advantages of TC. Long term persistence is the core factor that impacts the final outcome. More long-term follow-up studies (such as ≥ 2 years) are required, and positive approaches to promote patient long-term compliance via technology (such as apps, remote guidance) and community encourage require to be investigated.
